# Antibiotic prophylaxis after 48 h postoperatively are not associated with decreased surgical site infections and other healthcare associated infections in pancreatic surgery patients: a retrospective cohort study

**DOI:** 10.1186/s13756-023-01348-3

**Published:** 2023-12-02

**Authors:** Xiaoju Ma, Qiangsheng He, Youpeng Chen, Yan Lu, Ping Zhu, Ji Zhang, Wen-sen Chen, Yongxiang Zhang, Wei-hong Zhang, Chuanlong Zhu, Qiang Li, Zhanjie Li

**Affiliations:** 1 Department of Hospital Acquired Infection Control and Public Health Management, The Seventh Affiliated Hospital, Sun Yat-sen University, Shenzhen, Guangdong China; 2 Big Data Center, Scientific Research Center, The Seventh Affiliated Hospital, Sun Yat-sen University, Shenzhen, Guangdong China; 3 Clinical Research Center, The Seventh Affiliated Hospital, Sun Yat-sen University, Shenzhen, Guangdong China; 4 Department of Infectious Diseases, The Seventh Affiliated Hospital, Sun Yat-sen University, Shenzhen, Guangdong China; 5 Department of Medical Services, The First Affiliated Hospital of Nanjing Medical University, Nanjing, Jiangsu China; 6 Department of Pharmacy, The First Affiliated Hospital of Nanjing Medical University, Nanjing, Jiangsu China; 7 Department of Infection Control, The First Affiliated Hospital of Nanjing Medical University, No. 300 Guangzhou Road, Nanjing, 210029 Jiangsu China; 8 Department of Infections Disease, The First Affiliated Hospital of Nanjing Medical University, Nanjing, Jiangsu China; 9 Pancreas Center, The First Affiliated Hospital of Nanjing Medical University, No. 300 Guangzhou Road, Nanjing, 210029 Jiangsu China

**Keywords:** Antibiotic, Antibiotic prophylaxis after 48 h postoperatively, Pancreatic surgery, Surgical site infections, Healthcare-associated infections

## Abstract

**Background:**

It is controversial whether antibiotic should be used prophylactically 48 h after pancreatic surgery. Hence, the association of antibiotic prophylaxis (AP) after 48 h postoperatively with the incidence of surgical site infections (SSIs) and other healthcare-associated infections (HAIs) in patients receiving pancreatic surgery was evaluated.

**Methods:**

A retrospective cohort analysis was performed on 1073 patients who underwent pancreatic surgery. These patients were categorized into the non-AP after 48 h postoperatively group (n = 963) and the AP after 48 h postoperatively group (n = 110) based on whether or not they obtained AP from 48 h to 30 days after surgery. Outcomes included SSIs and other HAIs.

**Results:**

The incidence of SSIs in the non-AP after 48 h postoperatively group (98/963, 10.2%) was notably lower than that in the AP after 48 h postoperatively group (22/110, 20.0%) (*P* = 0.002). Other HAIs incidence was not significantly different between the non-AP after 48 h postoperatively group (77/963, 8.0%) and the AP after 48 h postoperatively group (11/110, 10.0%) (*P* = 0.468). Multiple regression analysis demonstrated that AP after 48 h postoperatively was a risk factor for SSIs (OR = 2.14, 95% CI 1.28–3.59) but not for other HAIs (OR = 1.24, 95% CI 0.63–2.42) after adjustment for age, gender, and diabetes. Subsequent to adjustment for all confounding factors, AP after 48 h postoperatively was not a influence factor for SSIs (OR = 2.13, 95% CI 0.76–5.99) and other HAIs (OR = 3.69, 95% CI 0.99–13.81).

**Conclusions:**

AP after 48 h postoperatively following pancreatic surgery was not associated with the lower morbidity rate of SSIs and other HAIs. Nonetheless, this study may facilitate further development of strategies towards standardization of the duration of AP management of pancreatic surgery.

## Introduction

Pancreatic surgery (including pancreaticoduodenectomy and distal pancreatectomy) is a complex and technically demanding treatment for patients with pancreatic diseases. With the development of surgical techniques and postoperative care, the perioperative mortality rate of patients undergoing pancreatic surgery has dropped to below 5% [1–3]. However, surgical site infections (SSIs) and other healthcare-associated infections (HAIs) remain great challenges for patients undergoing pancreatic surgery, which have high incidence rates and are pivotal factors of increased hospital readmission and mortality rates [4]. Several studies have demonstrated that the incidence of SSIs following pancreatic surgery was 11.58–26% [5–8], while that of other HAIs after pancreatectomy was 4.33–11.0% [9–11]. In addition, SSIs and other HAIs increase clinical burdens, prolong hospital stays, and elevate the costs of patients undergoing pancreatic surgery [5, 12]. Therefore, it is necessary to decrease the morbidity rate of HAIs after pancreatic surgery.

The microbiome of patients is tightly implicated in SSIs [13]. Post-pancreatic surgery infections frequently include infections with gram-positive, fungal, and drug-resistant organisms [14]. A prior study has shown that antibiotic prophylaxis (AP) reduces the incidence of SSIs [15]. In 2015, the *Chinese Guidelines for Clinical Use of Antibiotics* [16] classified that the duration of AP after clean-contaminated surgery and contaminated surgery should not exceed 24 h and might extend to 48 h for contaminated surgery when necessary. A recent study has elucidated that one preoperative antibiotic dose might be adequate for surgical prophylaxis in patients undergoing pancreatic surgery [17]. However, Fromentin et al. [18] and Hammad et al. [19] have reported that extended AP could reduce the incidence of SSI among high-risk patients. Excessive use of antibiotics can lead to the production of drug-resistant bacteria [20]. The microbes that cause SSIs have currently been unveiled to be resistant to antibiotics used for prophylaxis [21, 22]. Therefore, further research is warranted to clarify whether extended AP in patients receiving pancreatic surgery can diminish the incidence of SSIs and other HAIs.

In this study, the effect of AP after 48 h to 30 days postoperatively on the incidence of SSIs and other HAIs in patients undergoing pancreatic surgery were investigated.

## Participants and methods

### Study design

A retrospective cohort analysis was performed on all patients undergoing pancreatic surgery at the First Affiliated Hospital of Nanjing Medical University, a Grade-A tertiary hospital with 4500 beds. In this study, when the information system generates a clinician's order, the clinician has the option to specify the purpose of the medication, whether it is therapeutic or preventive. The empirical use of medication is a therapeutic purpose and is outside the scope of this study.

### Cohort construction

**Fig. 1:**
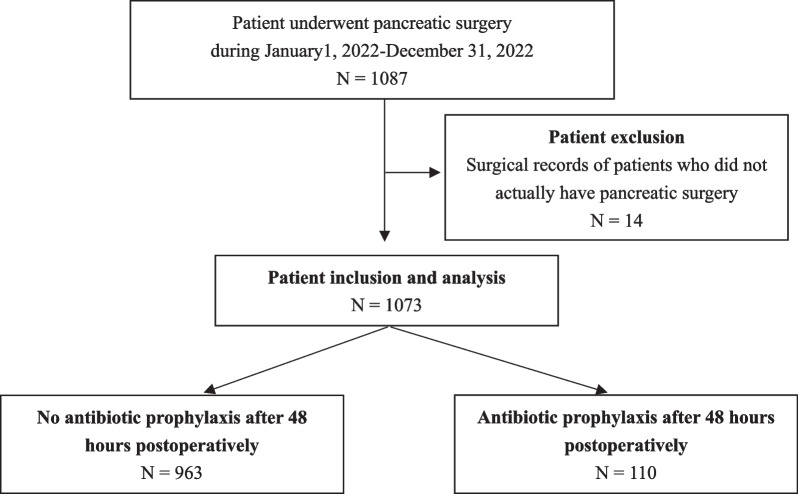
Flowchart of patient exclusion and inclusion

**Fig. 2:**
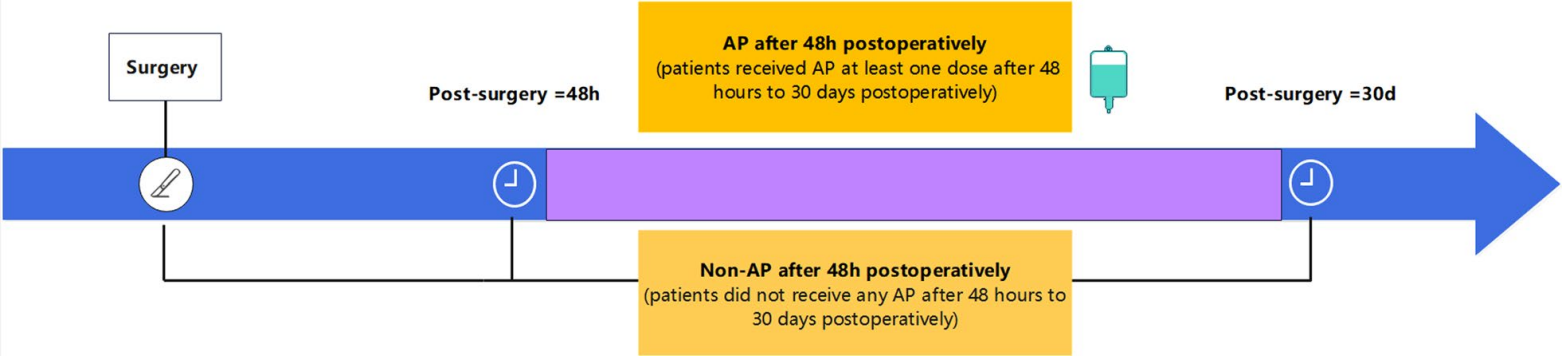
Range distinction between AP after 48 h postoperatively group and non-AP after 48 h postoperatively group

Patients undergoing the first pancreatic surgery from January 2022 to December 2022 were included in our study. The flowchart of this study is displayed in Fig. 1. A total of 14 patients who did not receive pancreatic surgery in the surgical records were excluded from 1087 patients. Finally, a total of 1073 patients were included and allocated into the non-AP after 48 h postoperatively group (n = 963; patients did not receive any AP after 48 h to 30 days postoperatively) and the AP after 48 h postoperatively group (n = 110; patients received AP at least one dose after 48 h to 30 days postoperatively). Two groups are distinguished in Fig. 2.

The Xinglin Real-Time Nosocomial Infection System and iih System were used to collect the demographic data of patients, including patient characteristics (age, sex, diabetes, and length of hospital stay), surgical variables [surgical category, surgical approach, surgical time, emergency, American Society of Anesthesiology (ASA) score (according to the principles of surgical risk assessment, I and II, score 0; III–V, score 1), National Nosocomial Infections Surveillance (NNIS) score, surgeon, inpatient department, and intraoperative blood loss], and Antibiotic (any AP, intraoperative redosing, AP within 48 h postoperatively, and AP days).

### Outcomes

Outcomes included SSIs (the primary outcome) within 30 days and other HAIs (the secondary outcome) [postoperative sepsis, postoperative pneumonia, pelvic and abdominal tissue infections (infections occur more than 30 days after surgery), and urinary tract infections, and others that occurred during the hospital stay after pancreatic surgery]. HAIs were diagnosed according to the Diagnostic Criteria of Nosocomial Infection (Trial) issued by the Ministry of Health in 2001 [23].

### Statistical analysis

Data were summarized as mean ± standard deviation (SD) or frequencies (percentages), as appropriate. The Chi-square or Fisher’s exact test and *t*-test or Mann–Whitney U test were utilized for descriptive statistics, as appropriate.

Continuous variables were classified into two categorical variables based on the SSI-risk age (65 years), the length of hospital stay of patients receiving pancreatic surgery (14 days), the 75% time of pancreatic surgery (4.88 h), and the days of prophylaxis (one day), respectively. Intraoperative blood loss was categorized into three categorical variables.

Univariate and multivariate logistic regression analyses were performed to assess the associations between AP after 48 h postoperatively and HAIs. The adjusted covariates in model 1 were age (continuous), sex (male or female), and diabetes (yes or no). In model 2, the adjusted covariates were age (continuous), sex (male or female), diabetes (yes or no), surgical category (pancreaticoduodenectomy, distal pancreatectomy, and others), surgical approach (non-endoscopic surgery and endoscopic surgery), surgical time (continuous), emergency (yes or no), ASA score (0 and 1), NNIS score 1 (0 and 1 point) and 2 (2 and 3 points), inpatient department (pancreatic center and nonpancreatic center), surgeon (Doctor 1, Doctor 2, Doctor 3, Doctor 4, Doctor 5, Doctor 6, and others), intraoperative blood loss (continuous), any AP (yes or no), intraoperative redosing (required but no redosing, required and redosing, or not required), AP within 48 h postoperatively (yes or no), and AP days (continuous).

A series of analyses were conducted to examine whether there was effect modification by age, sex, diabetes, surgical category, ASA score (0 and 1), NNIS score 1 (0 and 1 point) and 2 (2 and 3 points), inpatient department, surgical time, intraoperative blood loss, intraoperative redosing, and AP within 48 h postoperatively. For these analyses, we included an interaction term in the primary model between AP after 48 h postoperatively and these variables.

Analyses were performed using Statistical Product and Service Solutions (SPSS) (version 23.0; IBM Corp. Armonk, NY, USA), R software (version 3.6.0; R Core Team), EmpowerStats (www.empowerstates.com), and Graph Pad Prism 8.0 (San Diego, CA, USA). A *P* value of < 0.05 indicated a statistically significant difference.

## Results

### Patient characteristics, surgical variables, and outcomes

#### Patient characteristics

The mean (SD) age of patients at the time of hospitalization was 59.58 (13.29) years in the non-AP after 48 h postoperatively group and 62.97 (13.55) years in the AP after 48 h postoperatively group (*P* = 0.012)**.** No significant difference in other baseline characteristics (sex, diabetes, and length of hospital stay) between the two groups was found (Table 1).

**Table 1:** Descriptive data and outcomes of patients categorized by non-AP after 48 h postoperatively and AP after 48 h postoperatively

Characteristic	Non-AP after 48 h postoperatively (n = 963)	AP after 48 h postoperatively (n = 110)	*P* value
*Patient characteristics*			
Age (years)	59.58 ± 13.29	62.97 ± 13.55	0.012
Sex (male)	535 (55.6)	66 (60.0)	0.374
Diabetes	164 (17.0)	20 (18.2)	0.761
Length of hospital stay	22.90 ± 14.15	25.60 ± 13.23	0.057
*Surgical variables*			
Surgical category			0.472
Pancreaticoduodenectomy	481 (49.9)	61 (55.5)	
Distal pancreatectomy	315 (32.7)	30 (27.3)	
Others	167 (17.3)	19 (17.3)	
Surgical approach			0.303
Non-endoscopic surgery	833 (86.5)	99 (90.0)	
Endoscopic surgery	130 (13.5)	11 (10.0)	
Emergency	52 (5.4)	6 (5.5)	0.981
ASA score			0.006
0	766 (79.5)	75 (68.2)	
1	197 (20.5)	35 (31.8)	
NNIS score			0.018
1 (0 and 1 point)	819 (85.0)	84 (76.4)	
2 (2 and 3 points)	144 (15.0)	26 (23.6)	
Inpatient department			< 0.001
Non-pancreatic center	67 (7.0)	24 (21.8)	
Pancreatic center	896 (93.0)	86 (78.2)	
Surgeon			< 0.001
Doctor 1	65 (6.7)	5 (4.5)	
Doctor 2	156 (16.2)	15 (13.6)	
Doctor 3	411 (42.7)	41 (37.3)	
Doctor 4	73 (7.6)	14 (12.7)	
Doctor 5	47 (4.9)	4 (3.6)	
Doctor 6	140 (14.5)	6 (5.5)	
Others	71 (7.4)	25 (22.7)	
Surgical time (h)	3.97 ± 1.53	4.18 ± 1.70	0.195
Intraoperative blood loss (mL)	253.24 ± 451.43	329.00 ± 439.76	0.095
*Antibiotic*			
Any AP	821 (85.3)	110 (100)	< 0.001
Intraoperative redosing			0.531
Required but no redosing	657 (68.2)	76 (69.1)	
Required and redosing	34 (3.5)	6 (5.5)	
Not required	272 (28.2)	28 (25.5)	
AP within 48 h postoperatively	41 (4.3)	92 (83.6)	< 0.001
AP days	0.91 ± 0.52	6.95 ± 3.67	< 0.001
*Outcomes*			
SSIs	98 (10.2)	22 (20.0)	0.002
other HAIs	77 (8.0)	11 (10.0)	0.468

AP, antibiotic prophylaxis; ASA, American Society of Anesthesiology; NNIS, National Nosocomial Infections Surveillance; SSIs, surgical site infections; HAIs, healthcare-associated infections

#### Surgical variables

Among the surgical variables, ASA score 1 and NNIS score 1 (0 and 1 point) in the AP after 48 h postoperatively group was higher than that in the non-AP after 48 h postoperatively group (*P* = 0.006, *P* = 0.018). In addition, significant differences were found between the two groups in terms of inpatient department and surgeons (*P* < 0.001) (Table 1).

#### Outcomes

The incidence of SSIs in the non-AP after 48 h postoperatively group (98/963, 10.2%) was lower than that in the AP after 48 h postoperatively group (22/110, 20.0%) (*P* = 0.002). There was no significant difference between the incidence of other HAIs in the non-AP after 48 h postoperatively group (77/963, 8.0%) and the AP after 48 h postoperatively group (11/110, 10.0%) (*P* = 0.468) (Table 1).

### Analysis of AP

**Fig. 3:**
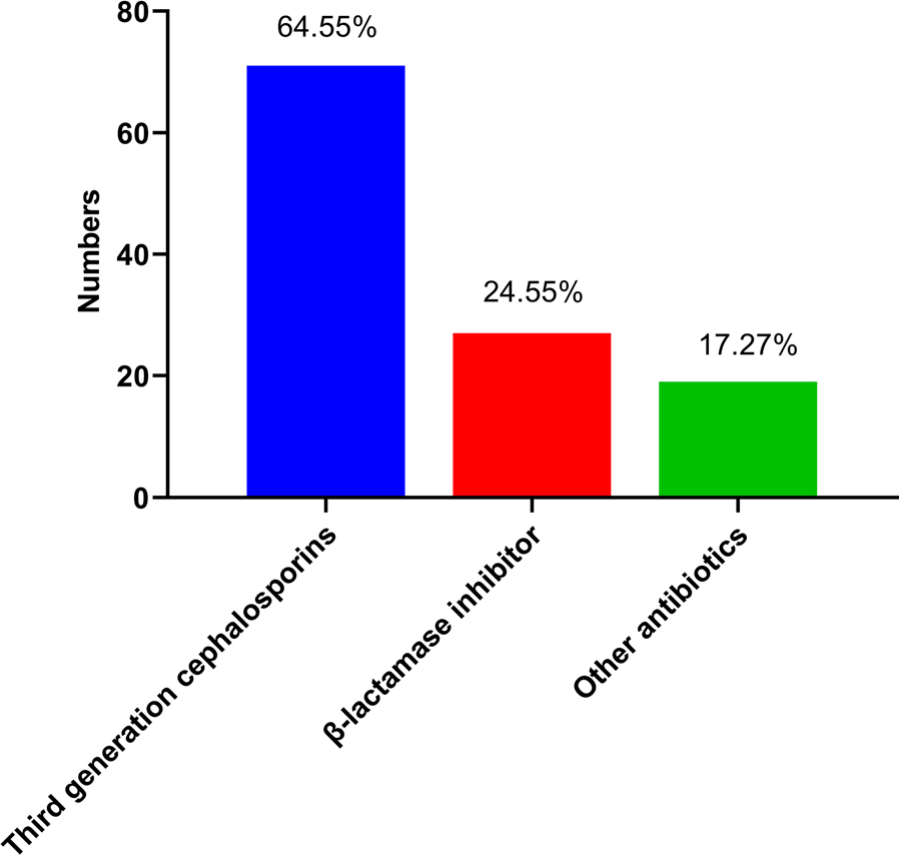
Types of prophylactic antibiotics in AP after 48 h postoperatively group

Any AP was used in 821 (85.3%) in the non-AP after 48 h postoperatively group, and 110 (100%) in the AP after 48 h postoperatively group (*P* < 0.001). The proportion of AP within 48 h postoperatively (83.6% vs. 4.3%) was higher in the AP after 48 h postoperatively group than that in the non-AP after 48 h postoperatively group (*P* < 0.001) (Table 1). The type of AP after 48 h postoperatively are depicted in Fig. 3, including third-generation cephalosporins, β-lactamase inhibitor, and other antibiotics (including Carbapenem antibiotic, Latamoxef, Fluoroquinolones, Clindamycin, and Fluconazole).

### AP after 48 h postoperatively and SSIs and other HAIs

According to the univariate logistic regression analysis results, the influence factors of SSIs included sex (female), length of hospital stay, surgical category (distal pancreatectomy), ASA score (0 and 1), NNIS score 1 (0 and 1 point) and 2 (2 and 3 points), surgical time, intraoperative blood loss, intraoperative redosing and AP within 48 h postoperatively (*P* < 0.05), while the influence factors of other HAIs included sex (female), length of hospital stay, surgical category (distal pancreatectomy), ASA score (0 and 1), surgical time, intraoperative blood loss, and any AP (*P* < 0.05) (Table 2).

**Table 2:** Univariate logistic regression for the association of suspected influence factors with SSIs and other HAIs

Exposure	SSIs		Other HAIs	
	OR (95% CI)	*P* value	OR (95% CI)	*P* value
*Patient characteristics*				
Age (years)	1.01 (0.99, 1.02)	0.359	1.01 (0.99, 1.02)	0.448
Sex				
Male	1		1	
Female	0.53 (0.35, 0.80)	0.002	0.60 (0.38, 0.95)	0.031
Diabetes				
No	1		1	
Yes	0.96 (0.58, 1.60)	0.882	1.47 (0.87, 2.49)	0.149
Length of hospital stay	1.06 (1.05, 1.08)	< 0.001	1.07 (1.06, 1.09)	< 0.001
*Surgical variables*				
Surgical category				
Pancreaticoduodenectomy	1		1	
Distal pancreatectomy	0.31 (0.18, 0.52)	< 0.001	0.28 (0.14, 0.54)	< 0.001
Others	0.47 (0.26, 0.83)	0.009	1.03 (0.60, 1.76)	0.928
Surgical approach				
Non-endoscopic surgery	1		1	
Endoscopic surgery	0.57 (0.29, 1.12)	0.102	0.46 (0.20, 1.08)	0.073
Emergency				
No	1		1	
Yes	1.71 (0.84, 3.48)	0.137	1.31 (0.55, 3.15)	0.542
ASA score				
0	1		1	
1	1.73 (1.14, 2.63)	0.010	1.68 (1.04, 2.72)	0.033
NNIS score				
1 (0 and 1 point)	1		1	
2 (2 and 3 points)	2.04 (1.31, 3.19)	0.002	1.52 (0.89, 2.60)	0.126
Inpatient department				
Nonpancreatic center	1		1	
Pancreatic center	0.61 (0.34, 1.09)	0.097	0.80 (0.39, 1.65)	0.540
Surgeon				
Doctor 1	1		1	
Doctor 2	2.23 (0.82, 6.07)	0.118	3.27 (0.73, 14.69)	0.122
Doctor 3	1.76 (0.68, 4.57)	0.243	2.85 (0.67, 12.14)	0.156
Doctor 4	1.32 (0.41, 4.22)	0.644	4.42 (0.93, 20.86)	0.061
Doctor 5	0.26 (0.03, 2.30)	0.226	3.70 (0.69, 19.87)	0.128
Doctor 6	1.16 (0.39, 3.44)	0.784	3.04 (0.66, 13.99)	0.153
Others	2.41 (0.83, 6.97)	0.105	3.52 (0.74, 16.82)	0.115
Surgical time (h)	1.40 (1.25, 1.57)	< 0.001	1.28 (1.13, 1.45)	< 0.001
Intraoperative blood loss (mL)	1.00 (1.00, 1.00)	0.029	1.00 (1.00, 1.00)	0.005
*Antibiotic*				
Any AP				
No	1		1	
Yes	1.17 (0.65, 2.11)	0.591	0.48 (0.28, 0.82)	0.007
Intraoperative redosing				
Required but no redosing	1		1	
Required and redosing	2.32 (1.10, 4.91)	0.027	0.81 (0.24, 2.68)	0.725
Not required	0.44 (0.26, 0.75)	0.002	0.63 (0.37, 1.09)	0.098
AP within 48 h postoperatively				
No	1		1	
Yes	1.94 (1.19, 3.16)	0.008	0.90 (0.45, 1.78)	0.759
AP days	1.07 (1.00, 1.15)	0.056	0.92 (0.81, 1.05)	0.236

SSIs, surgical site infections; HAIs, healthcare-associated infections; OR, Odds ration; CI, confidence interval; ASA, American Society of Anesthesiology; NNIS, National Nosocomial Infections Surveillance; AP, antibiotic prophylaxis

As demonstrated by the univariate logistic regression analysis results, AP after 48 h postoperatively was a risk factor of SSIs (OR = 2.21, 95% CI 1.32–3.68, *P* = 0.002) but not a risk factor of other HAIs (OR = 1.28, 95% CI 0.66–2.49, *P* = 0.469). The multivariate logistic regression analysis results revealed that subsequent to adjustment for the confounding effect of age, gender and diabetes were associated with SSIs (OR = 2.14, 95% CI 1.28–3.59, *P* = 0.004) but not with other HAIs (OR = 1.24, 95% CI 0.63–2.42, *P* = 0.532). Furthermore, after adjustment for all confounding factors, AP after 48 h postoperatively was not a influence factor for SSIs (OR = 2.13, 95% CI 0.76–5.99, *P* = 0.153) and other HAIs (OR = 3.69, 95% CI = 0.99–13.81, *P* = 0.053). The data are detailed in Table 3. The logistic regression analysis results for the covariate variables are displayed in Table 4.

**Table 3:** Associations of non-AP after 48 h postoperatively and AP after 48 h postoperatively with HAIs in patients undergoing pancreatic surgery

Exposure	Unadjusted estimate*		Model 1*	Adjusted estimate^1^	Model 2*	Adjusted estimate^2^
	OR (95% CI)	*P* value	OR (95% CI)	*P* value	OR (95% CI)	*P* value
*SSIs*						
Postoperative AP						
No	1		1		1	
Yes	2.21 (1.32, 3.68)	0.002	2.14 (1.28, 3.59)	0.004	2.13 (0.76, 5.99)	0.153
*Other HAIs*						
Postoperative AP						
No	1		1		1	
Yes	1.28 (0.66, 2.49)	0.469	1.24 (0.63, 2.42)	0.532	3.69 (0.99, 13.81)	0.053

AP, antibiotic prophylaxis; SSIs, surgical site infections; HAIs, healthcare-associated infections; OR, odds ration; CI, confidence interval; ASA, American Society of Anesthesiology; NNIS, National Nosocomial Infections Surveillance

^1^Model 1 was adjusted for age, sex, and diabetes

^2^Model 2 was adjusted for age, sex, diabetes, surgical category, surgical approach, surgical time, emergency, ASA score (0 and 1), NNIS score 1 (0 and 1 point) and 2 (2 and 3 points), surgeon, inpatient department, intraoperative blood loss, any AP, intraoperative redosing, AP within 48 h postoperatively, and AP days

*The sample size used for unadjusted estimate, Model 1, and Model 2 is all 1073

**Table 4:** Multivariate logistic regression analysis for the association of covariate variables with SSIs and other HAIs

Exposure	SSIs		Other HAIs	
	OR (95% CI)	*P* value	OR (95%C I)	*P* value
*Patient characteristics*				
Age (years)	0.99 (0.98, 1.01)	0.493	1.01 (0.99, 1.03)	0.462
Sex				
Male	1		1	
Female	0.66 (0.43, 1.02)	0.060	0.81 (0.49, 1.32)	0.388
Diabetes				
No	1		1	
Yes	0.81 (0.47, 1.40)	0.454	1.41 (0.80, 2.48)	0.235
*Surgical variables*				
Surgical category				
Pancreaticoduodenectomy	1		1	
Distal pancreatectomy	0.45 (0.25, 0.80)	0.007	0.32 (0.15, 0.68)	0.003
Others	0.59 (0.30, 1.17)	0.132	1.23 (0.62, 2.44)	0.559
Surgical approach				
Non-endoscopic surgery	1		1	
Endoscopic surgery	0.42 (0.17, 1.00)	0.050	0.44 (0.15, 1.25)	0.123
Emergency				
No	1		1	
Yes	1.66 (0.77, 3.60)	0.197	1.06 (0.40, 2.81)	0.906
ASA score				
0	1		1	
1	1.96 (0.73, 5.30)	0.185	2.26 (0.85, 6.01)	0.102
NNIS score				
1 (0 and 1 point)	1		1	
2 (2 and 3 points)	0.79 (0.27, 2.34)	0.675	0.42 (0.14, 1.29)	0.132
Inpatient department				
Non-pancreatic center	1		1	
Pancreatic center	0.72 (0.26, 2.01)	0.532	0.74 (0.24, 2.27)	0.603
Surgeon				
Doctor 1	1		1	
Doctor 2	2.67 (0.86, 8.27)	0.088	3.50 (0.70, 17.47)	0.126
Doctor 3	1.57 (0.58, 4.23)	0.374	2.56 (0.58, 11.17)	0.213
Doctor 4	1.24 (0.37, 4.13)	0.725	3.61 (0.73, 17.75)	0.114
Doctor 5	0.22 (0.02, 1.99)	0.178	3.76 (0.68, 20.90)	0.130
Doctor 6	1.18 (0.38, 3.64)	0.772	2.45 (0.51, 11.66)	0.261
Others	1.04 (0.25, 4.24)	0.958	1.70 (0.27, 10.59)	0.570
Surgical time (h)	1.28 (1.09, 1.49)	0.002	1.21 (1.01, 1.45)	0.037
Intraoperative blood loss (mL)	1.00 (1.00, 1.00)	0.984	1.00 (1.00, 1.00)	0.020
*Antibiotic*				
Any AP				
No	1		1	
Yes	1.10 (0.57, 2.12)	0.776	0.65 (0.33, 1.25)	0.192
Intraoperative redosing				
Required but no redosing	1		1	
Required and redosing	2.12 (0.92, 4.86)	0.077	0.91 (0.26, 3.23)	0.881
Not required	1.17 (0.55, 2.50)	0.686	1.15 (0.50, 2.62)	0.740
AP within 48 h postoperatively				
No	1		1	
Yes	1.26 (0.56, 2.85)	0.582	0.98 (0.31, 3.13)	0.978
AP days	0.95 (0.81, 1.11)	0.481	0.78 (0.58, 1.05)	0.098

SSIs, surgical site infections; HAIs, healthcare-associated infections; OR, odds ration; CI, confidence interval; ASA, American Society of Anesthesiology; NNIS, National Nosocomial Infections Surveillance; AP, antibiotic prophylaxis

### Subgroup analysis results

**Fig. 4:**
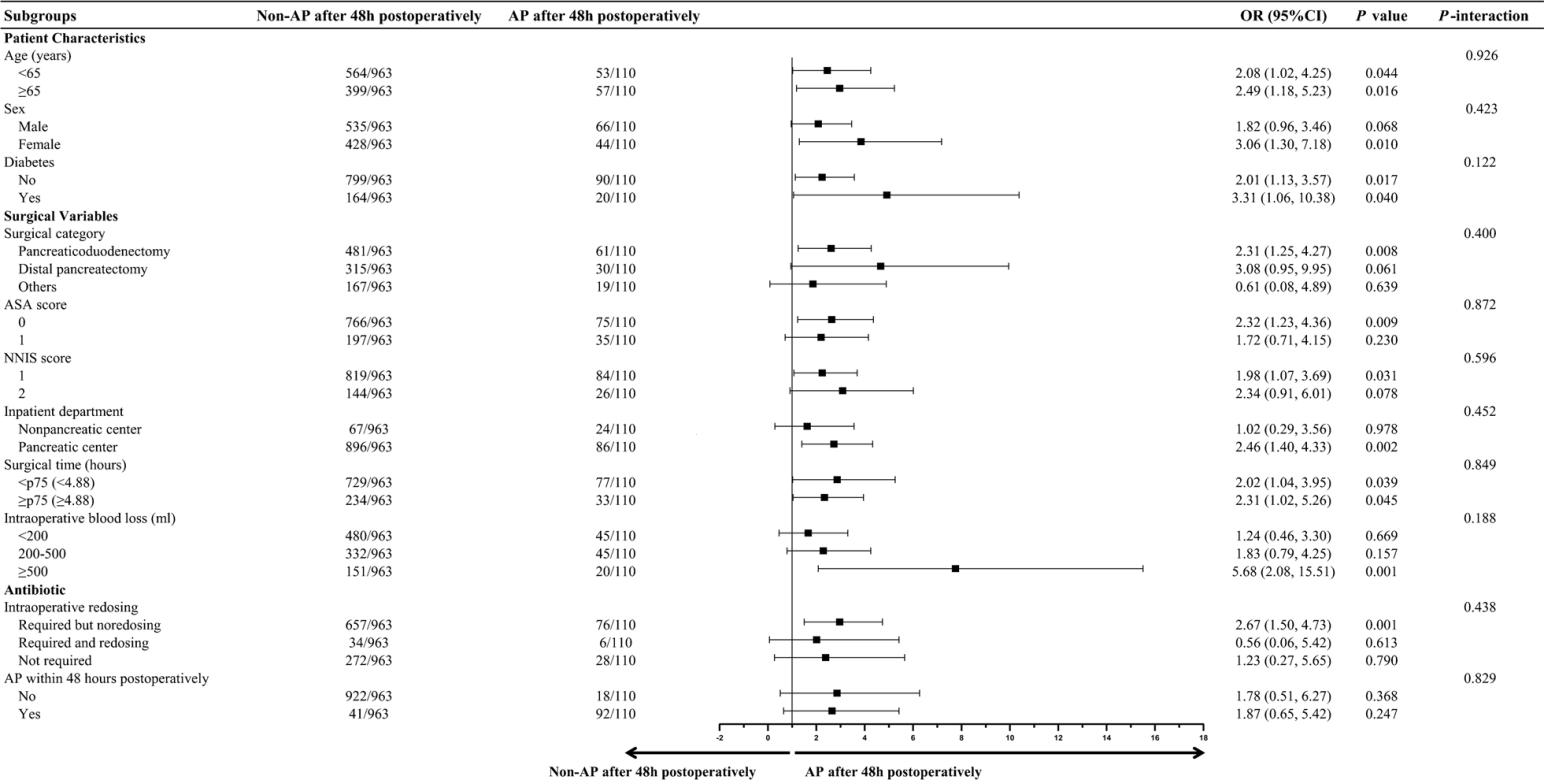
Subgroup analyses of SSIs according to non-AP after 48 h postoperatively and AP after 48 h postoperatively group

**Fig. 5:**
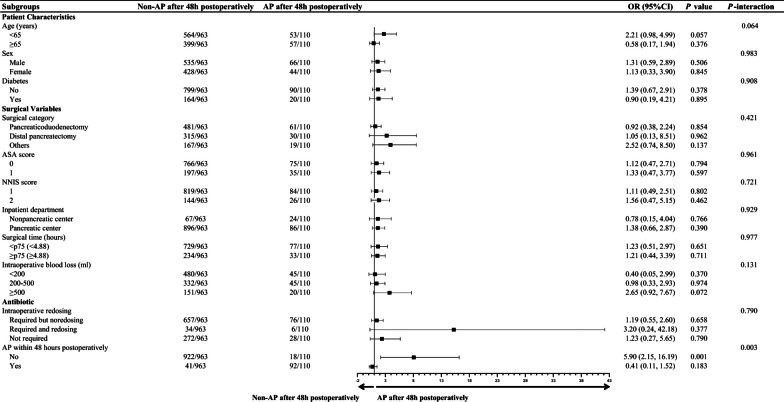
Subgroup analyses of other HAIs according to non-AP after 48 h postoperatively and AP after 48 h postoperatively group

Subgroup analyses showed that the estimated risk of SSIs with AP after 48 h postoperatively did not differ by age, sex, diabetes, surgical category, ASA score (0 and 1), NNIS score 1 (0 and 1 point) and 2 (2 and 3 points), inpatient department, surgical time, intraoperative blood loss, intraoperative redosing, and AP within 48 h postoperatively (Fig. 4). Additionally, the estimated risk of other HAIs with AP after 48 h postoperatively did not differ by age, sex, diabetes, surgical category, ASA score (0 and 1), NNIS score 1 (0 and 1 point) and 2 (2 and 3 points), inpatient department, surgical time, intraoperative blood loss, and intraoperative redosing, but by AP within 48 h postoperatively (Fig. 5).

## Discussion

In this study, AP after 48 h postoperatively with third-generation cephalosporins, β-lactamase inhibitor, or other antibiotics did not statistically and clinically significantly diminish the prevalence of SSIs in patients undergoing pancreatic surgery. Additionally, AP after 48 h postoperatively was not associated with the decreased morbidity rate of other HAIs (including postoperative sepsis, postoperative pneumonia, pelvic and abdominal tissue infections, and urinary tract infections).

Postoperative infections can cause antibiotic overuse [24]. Therefore, a preventive anti-infectious strategy is needed to reduce the postoperative risk of HAIs and to avoid prolonged antibiotic exposure. Current guidelines in China recommend that antibiotic use after clean-contaminated and contaminated surgery should be discontinued within 24 h after the end of surgery and extended to 48 h for contaminated hepatopancreatobiliary surgery if necessary [16]. Similarly, the *American Clinical Practice Guidelines for Antimicrobial Prophylaxis in Surgery* recommend that prophylaxis for the duration of the procedure and certainly for less than 24 h is appropriate [25]. However, these are inconsistent with the requirements and implementation of AP for pancreatic surgery. Macedo et al. [26] have reported that 69.47% of 285 hepatopancreatobiliary surgeons extended AP beyond 3 days worldwide. In our research, AP was conducted in 10.25% of 1073 patients receiving pancreatic surgery after 48 h postoperatively, and the duration of AP after 48 h postoperatively was 6.95 ± 3.67 days. The reason for the above situation is that the cognition of the duration of AP in patients receiving pancreatic surgery remains controversial. Recent evidence has unraveled that longer (72 h) broad-spectrum antibiotic coverage notably lowers the incidence of SSIs after pancreaticoduodenectomy surgery when compared with routine use (24 h) [27]. Similarly, some prior studies have elaborated that extended antibiotic use is correlated with the reduced incidence rate of SSIs following pancreatoduodenectomy among high-risk patients [18, 19]. As evidenced by a systematic review and meta-analysis involving ten studies, prolonged antibiotic prophylaxis between 2 and 10 days after pancreatoduodenectomy is associated with fewer organ/space infections in patients who undergo preoperative biliary drainage [28]. On the contrary, a systematic review has illustrated that a single preoperative dose of cefazolin for hepatopancreatobiliary surgery is indicated for AP [17]. In our study, we revealed an insignificant correlation between all types of SSIs and other HAIs with AP after 48 h postoperatively after adjustment for the confounding factors of patients. This supports the Chinese and American guidelines.

The excessive or frequent prescription of antibiotics may not reduce the incidence of postoperative infections at all, which may even oppositely increase bacterial resistance to trigger multiple infections [20]. Therefore, rational duration of perioperative AP is of great importance and necessity. In addition to the duration of AP, the selection of antibiotics is also highly critical for diminishing the rate of postoperative infections. As reported, bacterial colonization in the surgical site is closely associated with the occurrence of SSIs in patients undergoing pancreatic surgery; it is necessary to conduct targeted AP covering microbes prevalent in post-pancreatic surgery infections [29, 30]. Chinese guidelines recommend the administration of first- and second-generation cephalosporin or ceftriaxone with or without metronidazole, as well as cephalomycin, as perioperative AP in hepatopancreatobiliary surgery [16]. However, the selection of antibiotics is widely heterogeneous, such as first-generation cephalosporin/metronidazole, second-generation cephalosporin, ciprofloxacin/metronidazole, ampicillin/sulbactam (Unasyn), ampicillin/gentamicin/metronidazole, and extended-spectrum penicillin [25, 26]. The results of the present study revealed an inconsistency in the type of antibiotics used for AP after 48 h postoperatively in pancreatic surgery, including third-generation cephalosporins, β-lactamase inhibitor, and other antibiotics. These findings were also inconsistent with the guideline. The main reason for the prophylactic use of certain broad-spectrum antibiotics may be the escalation of antibiotic usage by clinicians who perceive a higher infection rate after pancreatic surgery, particularly pancreaticoduodenectomy. The use of high-level antibiotics (such as third-generation cephalosporins and Carbapenem antibiotic) may be due to the expansion of antimicrobial resistance [31, 32]. Importantly, the common drug-resistant bacteria are a cause of SSIs and other HAIs [22, 33]. Accordingly, clinicians should closely monitor patients and select proper antibiotics.

The present study has some strengths. First, existing studies mainly focused on the specific or selected populations; however, in this study, the whole population of patients undergoing pancreatic surgery was included. Although these results were derived from the data of a single center, our sample size was large enough to exceed 1000. Therefore, these findings are applicable to real-world situations. Second, a subgroup analysis was performed and three unadjusted and adjusted models (adjusted for confounding factors) were constructed in our study, which emphasizes the credibility of our results. Third, in addition to the association between AP after 48 h postoperatively and the incidence of SSIs, this study also highlighted the effect of other HAIs. Nevertheless, there are some limitations in the present study. Our data were collected from a single-center study, and more relevant factors should be further analyzed, such as body mass index, preoperative administration time, drain placement, and malnutrition. In addition, our study may involve some subjective factors of surgeons, who overuse AP because of suspecting the patient with a greater risk of infection, suspected infection, underestimation of infection [34] or prescribing preventive medications as treatments, which cannot be reflected in the objective factors and cannot be corrected.

## Conclusions

This study revealed no statistically significant decrease in the incidence of SSIs and other HAIs in patients receiving postoperative AP. Nonetheless, this study may facilitate further development of strategies towards standardization of the duration of AP management of pancreatic surgery. These findings indicate that in addition to focusing on the duration of postoperative AP, the adaptation of antimicrobial prophylaxis should also be evaluated according to pancreatic surgery performance and local epidemiology to avoid the overuse of antibiotics.

## Data Availability

All data generated or analyzed during this study are included in this published article.

## Abbreviations

AP: Antibiotic prophylaxis
SSIs: Surgical site infections
HAIs: Healthcare-associated infections
ASA: American Society of Anesthesiology
NNIS: National Nosocomial Infections Surveillance
OR: Odds ration
CI: Confidence interval

## References

[1] Kimura W, Miyata H, Gotoh M (2014). A pancreaticoduodenectomy risk model derived from 8575 cases from a national single-race population (Japanese) using a web-based data entry system: the 30-day and in-hospital mortality rates for pancreaticoduodenectomy. Ann Surg.

[2] Wang XX, Yan YK, Dong BL (2021). Pancreatic outflow tract reconstruction after pancreaticoduodenectomy: a meta-analysis of randomized controlled trials. World J Surg Oncol.

[3] Tang W, Zhang YF, Zhao YF (2022). Comparison of laparoscopic versus open radical antegrade modular pancreatosplenectomy for pancreatic cancer: a systematic review and meta-analysis. Int J Surg.

[4] Peluso H, Jones WB, Parikh AA (2019). Treatment outcomes, 30-day readmission and healthcare resource utilization after pancreatoduodenectomy for pancreatic malignancies. J Hepatobiliary Pancreat Sci.

[5] De Pastena M, Paiella S, Fontana M (2022). The clinical and economic impact of surgical site infections after distal pancreatectomy. Surgery.

[6] Nobuhara H, Matsugu Y, Tanaka J (2022). The preventive effects of perioperative oral care on surgical site infections after pancreatic cancer surgery: a retrospective study. Support Care Cancer.

[7] Pollini T, Wong P, Kone LB (2023). Drain placement after pancreatic resection: friend or foe for surgical site infections?. J Gastrointest Surg.

[8] Mentor K, Ratnayake B, Akter N (2020). Meta-analysis and meta-regression of risk factors for surgical site infections in hepatic and pancreatic resection. World J Surg.

[9] Lee DU, Hastie DJ, Fan GH (2022). Effect of malnutrition on the postoperative outcomes of patients undergoing pancreatectomy for pancreatic cancer: propensity score-matched analysis of 2011–2017 US hospitals. Nutr Clin Pract.

[10] Yang PS, Liu CP, Hsu YC (2019). A novel prediction model for bloodstream infections in hepatobiliary-pancreatic surgery patients. World J Surg.

[11] Okada K, Uemura K, Ohge H (2022). Prognostic impact of postoperative infection in patients with pancreatic cancer: a multicenter cohort study. Surgery.

[12] Fadayomi AB, Kasumova GG, Tabatabaie O (2018). Unique predictors and economic burden of superficial and deep/organ space surgical site infections following pancreatectomy. HPB (Oxford).

[13] Alverdy JC, Hyman N, Gilbert J (2020). Re-examining causes of surgical site infections following elective surgery in the era of asepsis. Lancet Infect Dis.

[14] Behrman SW, Bahr MH, Dickson PV (2011). The microbiology of secondary and postoperative pancreatic infections: implications for antimicrobial management. Arch Surg.

[15] De Pastena M, Paiella S, Azzini AM (2021). Antibiotic prophylaxis with piperacillin-tazobactam reduces post-operative infectious complication after pancreatic surgery: an interventional, non-randomized study. Surg Infect (Larchmt).

[16] Circular on the issuance of the guiding principles for the clinical application of antimicrobial drugs (2015 edition). Bulletin of the National Health and Family Planning Commission of the People's Republic of China. 2015, No. 144(07): 29.

[17] Steccanella F, Amoretti P, Barbieri MR (2022). Antibiotic prophylaxis for hepato-biliopancreatic surgery-a systematic review. Antibiotics (Basel).

[18] Fromentin M, Mullaert J, Gille B (2022). Extended antibiotic prophylaxis after pancreatoduodenectomy reduces postoperative abdominal infection in high-risk patients: results from a retrospective cohort study. Surgery.

[19] Hammad AY, Khachfe HH, AlMasri S (2023). Impact of extended antibiotic use after pancreaticoduodenectomy for patients with preoperative metallic biliary stenting treated with neoadjuvant chemotherapy. J Gastrointest Surg.

[20] Medina E, Pieper DH (2016). Tackling threats and future problems of multidrug-resistant bacteria. Curr Top Microbiol Immunol.

[21] Teillant A, Gandra S, Barter D (2015). Potential burden of antibiotic resistance on surgery and cancer chemotherapy antibiotic prophylaxis in the USA: a literature review and modelling study. Lancet Infect Dis.

[22] Stecca T, Nistri C, Pauletti B (2020). Bacteriobilia resistance to antibiotic prophylaxis increases morbidity after pancreaticoduodenectomy: a monocentric retrospective study of 128 patients. Updates Surg.

[23] The Diagnostic Criteria of Nosocomial Infection (Trial). Zhong Hua Yi Xue Za Zhi. 2001(05): 61–67.

[24] Bortolotti P, Delpierre C, Le Guern R (2021). High incidence of postoperative infections after pancreaticoduodenectomy: a need for perioperative anti-infectious strategies. Infect Dis Now.

[25] Bratzler DW, Dellinger EP, Olsen KM (2013). Clinical practice guidelines for antimicrobial prophylaxis in surgery. Surg Infect (Larchmt).

[26] Macedo F, Mowzoon M, Parikh J (2017). Disparities in the management and prophylaxis of surgical site infection and pancreatic fistula after pancreatoduodenectomy. J Hepatobiliary Pancreat Sci.

[27] Fathi AH, Jackson T, Barati M (2016). Extended perioperative antibiotic coverage in conjunction with intraoperative bile cultures decreases infectious complications after pancreaticoduodenectomy. HPB Surg.

[28] Droogh D, Groen JV, de Boer M (2023). Prolonged antibiotic prophylaxis after pancreatoduodenectomy: systematic review and meta-analysis. Br J Surg.

[29] Pham H, Chen A, Nahm CB (2022). The role of targeted versus standard antibiotic prophylaxis in pancreatoduodenectomy in reducing postoperative infectious complications: a systematic review and meta-analysis. Ann Surg.

[30] Krüger CM, Adam U, Adam T (2019). Bacterobilia in pancreatic surgery-conclusions for perioperative antibiotic prophylaxis. World J Gastroenterol.

[31] Brink AJ (2019). Epidemiology of carbapenem-resistant Gram-negative infections globally. Curr Opin Infect Dis.

[32] Mizrahi A, Delerue T, Morel H (2020). Infections caused by naturally AmpC-producing Enterobacteriaceae: Can we use third-generation cephalosporins? A narrative review. Int J Antimicrob Agents.

[33] Bilgiç Ç, Keske Ş, Sobutay E (2020). Surgical site infections after pancreaticoduodenectomy: preoperative biliary system interventions and antimicrobial prophylaxis. Int J Infect Dis.

[34] Khan Z, Ahmed N, Zafar S (2020). Audit of antibiotic prophylaxis and adherence of surgeons to standard guidelines in common abdominal surgical procedures. East Mediterr Health J.

